# Cellular and Molecular Effects of High-Molecular-Weight Heparin on Matrix Metalloproteinase 9 Expression

**DOI:** 10.3390/ijms20071595

**Published:** 2019-03-30

**Authors:** René Huber, Rozan Attili/Abedalkhader, Daniela Küper, Lara Hauke, Bernadette Lüns, Korbinian Brand, Karin Weissenborn, Ralf Lichtinghagen

**Affiliations:** 1 Institute of Clinical Chemistry, Hannover Medical School, 30625 Hannover, Germany; huber.rene@mh-hannover.de (R.H.); rozana@hebron.edu (R.A.); kueper.daniela@mh-hannover.de (D.K.); lara.hauke@gmx.de (L.H.); luens.bernadette@mh-hannover.de (B.L.); brand.korbinian@mh-hannover.de (K.B.); 2 Central Pharmacy, Hannover Medical School, 30625 Hannover, Germany; 3 Department of Neurology, Hannover Medical School, 30625 Hannover, Germany; weissenborn.karin@mh-hannover.de

**Keywords:** blood sampling, anticoagulants, MMP-9, high-molecular-weight heparin, IL-16, sICAM-1, IL-8, monocytes, T cells

## Abstract

Blood sampling with different anticoagulants alters matrix metalloproteinase (MMP-) 9 expression, thus influencing its concentration and diagnostic validity. Here, we aimed to evaluate the effects of different anticoagulants on MMP-9 regulation. MMP-9 expression was assessed in response to ethylenediaminetetraacetic acid, citrate, and high-/low-molecular-weight heparin (HMWH, LMWH) in co-culture experiments using THP-1, Jurkat, and HT cells (representing monocytes, T, and B cells). Triple and double cell line co-culture experiments revealed that HMWH treatment of THP-1 and Jurkat led to a significant MMP-9 induction, whereas other anticoagulants and cell type combinations had no effect. Supernatant of HMWH-treated Jurkat cells also induced MMP-9 in THP-1 suggesting monocytes as MMP-9 producers. HMWH-induced cytokine/chemokine secretion was assessed in co-culture supernatant, and the influence of cytokines/chemokines on MMP-9 production was analyzed. These experiments revealed that Jurkat-derived IL-16 and soluble intercellular adhesion molecule (sICAM-) 1 are able to induce MMP-9 and IL-8 production by THP-1. As a consequence, the increased MMP-9 expression found in HMWH blood samples may be influenced by HMWH-dependent secretion of IL-16 and sICAM-1 by T cells resulting in an increased production of MMP-9 and IL-8 by monocytes. IL-8, in turn, may support MMP-9 and its own expression in a positive autocrine feedback loop.

## 1. Introduction

Matrix metalloproteinases (MMPs) are a major group of endopeptidases degrading extracellular matrix (ECM) and basement membrane components [1] involved in the regulation of cellular events such as proliferation, differentiation, and migration [2]. In humans, 23 zinc-dependent MMPs have been identified [3]. Emerging evidence indicate an important role of these enzymes in normal and pathological processes including tissue remodeling, wound healing, inflammation, arthritis, cardiovascular diseases, cancer, and neurological diseases [3,4,5]. Of particular importance is MMP-9, also known as gelatinase B or type IV collagenase, since it has recently become a subject of growing interest in diverse human pathologies such as stroke. Stroke is regarded as the one of the leading causes of death in the world [6] and MMP-9 levels in the blood have been suggested to represent a suitable biomarker supporting its prognosis [7]. It was previously shown by our group and others that the way of blood sampling may affect subsequent analytical tests and that the kind of anticoagulant used, especially heparin, may alter the amount of MMP-9 (and other proteases or protease inhibitors) measured [8,9,10,11]. The molecular mechanism underlying this phenomenon, however, is still not well elucidated. Nevertheless, currently, MMP-9 assessment in blood is performed using heparin, ethylenediaminetetraacetic acid (EDTA), citrate, or serum samples [12]. The clinical chemist who has to evaluate a patient’s blood MMP-9 concentration usually does not know about the patient’s medical treatment, and an impact of concurrent treatment upon measured MMP-9 levels has not been considered in practice, so far.

High-molecular-weight heparin (HMWH) is a highly sulfated glycosaminoglycan. Due to its high negative charge density, HMWH prevents clotting and is widely used as an anticoagulant in heparin blood samples [13]. Low-molecular-weight heparin (LMWH), another class of anticoagulant derived from unfractionated (i.e., high-molecular-weight) heparin, has a number of advantages over HMWH, which has led to its increasing use for a number of thromboembolic indications [13,14]. The interaction of HMWH, LMWH, and other commonly used anticoagulants such as EDTA [15] and citrate [16] with human blood cells and their potential modulation of cellular processes are not well defined, neither in general nor in respect of MMP-9 expression [17].

The aim of this study was to characterize the influence of direct and indirect effects of different anticoagulants on the regulation of MMP-9 and to identify the molecular mechanism mediating these effects so as to assess the impact of the respective substances on the suitability of MMP-9 as a biomarker.

## 2. Results

### 2.1. Direct Stimulation of Different Cell Types with Anticoagulants Has No Influence on MMP-9 Expression

**Figure 1:**
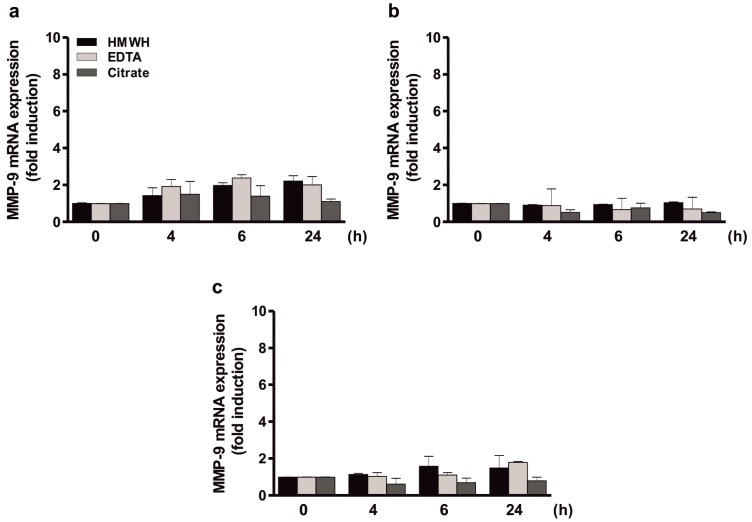
No significant induction of MMP-9 expression by HMWH, EDTA, or citrate in individual cell line cultures of THP-1, Jurkat, and HT cells. 2 × 10^5^ THP-1 (**a**), Jurkat (**b**), or HT cells (**c**) per well were starved overnight and then stimulated with 50 IU/well HMWH, 3.2 mg/well EDTA, or 220 μL/well citrate up to 24 h. MMP-9 mRNA expression was determined using qPCR (QuantiTect Custom Assay; housekeeping gene: GAPDH); mean ± SD, *n* = 3.

Stimulation of individual cell line cultures (THP-1, Jurkat, HT cells) with 3.2 mg/well EDTA (dissolved in medium), 220 µL/well citrate, or 50 international units (IU)/well HMWH revealed that MMP-9 expression and secretion are not influenced directly by any of these anticoagulants in monocytic cells (Figure 1a), T cells (Figure 1b), or B cells (Figure 1c) since no significant induction of MMP-9 mRNA could be shown by qPCR. This indicates that the reported MMP-9 induction in heparin blood sampling [8] is mediated via an indirect mechanism.

### 2.2. Significant Induction of MMP-9 Expression by HMWH in A Co-Culture Including THP-1, Jurkat, and HT Cells

**Figure 2:**
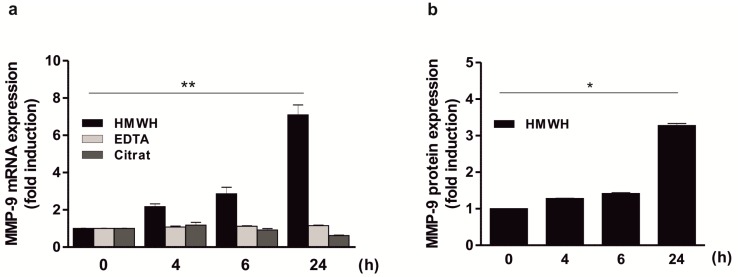
Induction of MMP-9 expression by HMWH in a THP-1, Jurkat, and HT cells containing co-culture. 7 × 10^5^ THP-1, Jurkat, and HT cells per well each (i.e., a total of 2.1 × 10^6^ cells/well) were starved overnight and then stimulated with 50 IU/well HMWH, 3.2 mg/well EDTA, or 220 μL/well citrate up to 24 h. MMP-9 mRNA (**a**) and protein (**b**) expression were determined using qPCR (QuantiTect Custom Assay; housekeeping gene: GAPDH) and ELISA (MMP-9 Quantikine Kit); mean ± SD, *n* = 3 (measured in duplicates). Kruskal–Wallis test, * *p* ≤ 0.05; ** *p* ≤ 0.01.

The analysis of MMP-9 mRNA expression in a co-culture of THP-1, Jurkat, and HT cells demonstrated that MMP-9 expression increased significantly over time after addition of HMWH (approximately 7-fold after 24 h; Figure 2a). Equivalent results were obtained when the amounts of secreted MMP-9 protein were measured in the culture supernatant (approximately 3.5-fold induction after 24 h; Figure 2b). In contrast, stimulation with other anticoagulants such as EDTA or citrate (Figure 2a) had no MMP-9-inducing effect in this co-culture model. These results suggest that both MMP-9 mRNA and protein expression in one of the cell types used depends on an interaction with another cell type present in the mixture in response to HMWH, potentially by cell-to-cell contacts or indirectly via the stimulation with a secreted mediator.

### 2.3. Significant Induction of MMP-9 Expression by HMWH in the THP-1 and Jurkat Co-Culture

**Figure 3:**
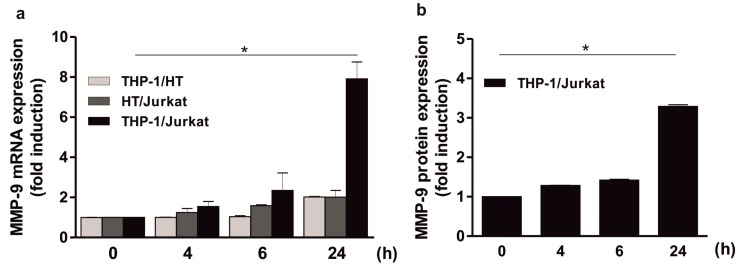
Induction of MMP-9 expression by HMWH in a THP-1 and Jurkat cells containing co-culture. 1 × 10^6^ THP-1 and HT, HT and Jurkat, or THP-1 and Jurkat cells per well (i.e., a total of 2 × 10^6^ cells/well) were starved overnight and then stimulated with 50 IU/well HMWH up to 24 h. MMP-9 mRNA (**a**) and protein (**b**) expression were determined using qPCR (QuantiTect Custom Assay; housekeeping gene: GAPDH) and ELISA (MMP-9 Quantikine Kit); mean ± SD, *n* = 3 (measured in duplicates). Kruskal–Wallis test, * *p* ≤ 0.05.

To determine whether the impact of HMWH on MMP-9 expression depends on an interplay of the three cell types used or a cooperation of two cell lines, MMP-9 expression was further assessed in co-culture approaches consisting of two cell types each. Thus, cell line mixtures including HMWH-stimulated monocytes and T cells, monocytes and B cells, as well as T and B cells were performed. In control approaches, the mixtures were alternatively treated with EDTA or citrate. As expected, no increase in MMP-9 mRNA expression was observed in the cultures of THP-1 and Jurkat, THP-1 and HT, as well as Jurkat and HT cells following control stimulation with EDTA or citrate (data not shown). Moreover, there was also no significant induction of MMP-9 levels following HMWH stimulation in a mixture of THP-1 and HT cells or HT and Jurkat cells (Figure 3a). In contrast, HMWH stimulation of a combination of THP-1 and Jurkat cells led to a significantly increased MMP-9 mRNA expression over time (approximately 8-fold after 24 h; Figure 3a). These results could also be confirmed on the protein level (approximately 3-fold induction after 24 h; Figure 3b).

### 2.4. Significant Induction of MMP-9 Expression in THP-1 Cells in Response to Culture Supernatant Derived from HMWH-Treated Jurkat Cells

**Figure 4:**
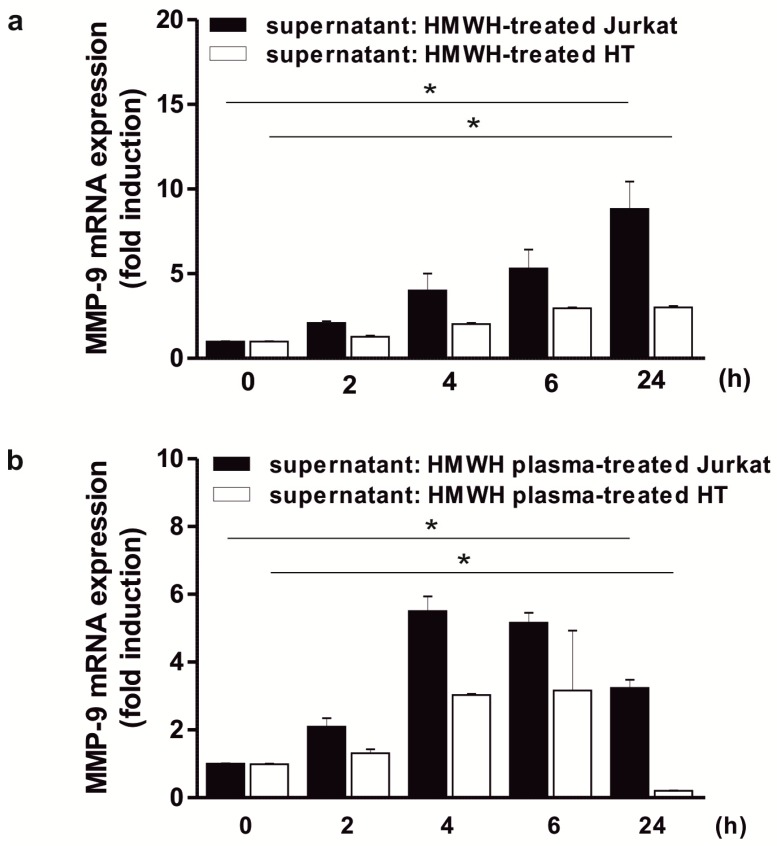
Induction of MMP-9 expression in THP-1 cells by incubation with supernatant from HMWH- and HMWH plasma-stimulated Jurkat cells. 2 × 10^6^ THP-1 cells/well were starved overnight and subsequently stimulated up to 24 h with the supernatant of Jurkat or HT cells which have been treated for 24 h with 50 IU/well HMWH (**a**) or HMWH plasma (derived from a heparin monovette) (**b**). MMP-9 mRNA expression was determined using qPCR (QuantiTect Custom Assay; housekeeping gene: GAPDH); mean ± SD, *n* = 3 (measured in duplicates). Kruskal–Wallis test, * *p* ≤ 0.05.

In the next step, it was analyzed whether the increased MMP-9 production depends on cell-to-cell-contacts or is rather based on an indirect effect, e.g., via soluble mediator(s). Since monocytes/macrophages are able to produce large amounts of MMP-9 [18], we hypothesized that in this context, the T cells are responsible for the secretion of soluble factor(s) activating the MMP-9 production in monocytes. Therefore, the MMP-9 expression in THP-1 cells following incubation with culture supernatant derived from HMWH-stimulated Jurkat cells was analyzed. In the respective experiments, a significant induction of MMP-9 mRNA up to 24 h could be shown (approximately 8-fold after 24 h) whereas the supernatant of HMWH-treated HT cells had only a slight (though significant) effect on MMP-9 (up to 3-fold; Figure 4a). Stimulation of THP-1 cells with the supernatant of EDTA- or citrate treated Jurkat and HT cells did not increase MMP-9 mRNA expression considerably (data not shown). THP-1 cells were also incubated with the supernatant from Jurkat cells which have been incubated with human plasma derived from heparin-containing monovettes and MMP-9 was also significantly increased in this experimental setting, albeit to a lesser extent and characterized by a divergent temporal development (peaking at 4 h with approximately 5.5-fold induction; Figure 4b). These data support the suggestion that the monocytes are the main producers of MMP-9 and that factor(s) present in the supernatant of HMWH-stimulated T cells are able to significantly induce MMP-9 expression in monocytes.

### 2.5. High-Molecular-Weight Heparin Versus Low-Molecular-Weight Heparin

**Figure 5:**
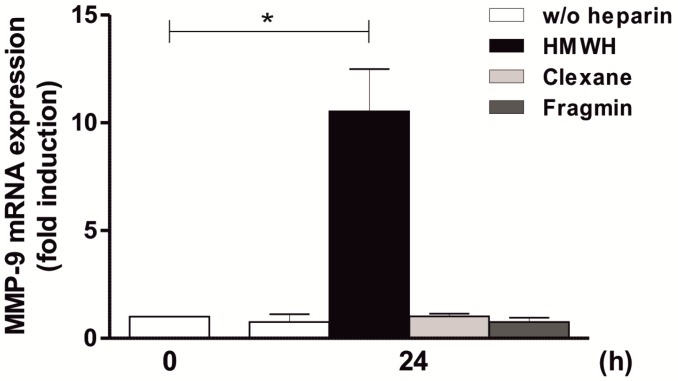
No Induction of MMP-9 expression by LMWH in a THP-1 and Jurkat cells containing co-culture. 1 × 10^6^ THP-1 and Jurkat cells (i.e., a total of 2 × 10^6^ cells/well) were starved overnight. Afterwards, the co-culture was stimulated with HMWH (as a positive control), Clexane, or Fragmin (50 IU/well each) for 24 h. Then, MMP-9 mRNA expression was determined using qPCR (QuantiTect Custom Assay; housekeeping gene: GAPDH); mean ± SD, *n* = 3 (measured in duplicates). T test, * *p* ≤ 0.05.

To further investigate the stimulatory effect of other types of heparin on MMP-9 expression, the effect of two different types of LMWH (i.e., Clexane and Fragmin) was analyzed. As demonstrated in Figure 5, the stimulation of THP-1 and Jurkat with LMWH had no increasing effect on MMP-9 mRNA amounts, neither using Clexane nor Fragmin, in contrast to the significant approximately 10-fold effect shown by HMWH after 24 h. These results indicate that induction of MMP-9 expression also depends on the type of heparin used.

### 2.6. Identification of Soluble Mediators Secreted in Response to HMWH

The basic idea of the following experiment was to identify the (combination of) T cell-derived mediator(s) which is able to increase MMP-9 synthesis in monocytes. Therefore, all three individual cell lines as well as the relevant double (THP-1 and Jurkat) and triple (THP-1, Jurkat, and HT) cell line mixtures were cultivated and stimulated with HMWH. Cell culture supernatants were then profiled for the expression of multiple cytokines and chemokines. As shown in Table 1, HMWH-stimulated THP-1 released 3 types of mediators, macrophage migration inhibitory factor (MIF), interleukin 1 receptor antagonist (IL-1RA), and CC-chemokine ligand 5 (CCL5). Jurkat secreted IL-13, IL-16, MIF, sICAM-1, and Serpin E1, whereas HT cells produced IL-13, MIF, tumor necrosis factor (TNF), SICAM-1, and Serpin E1. In the co-cultures of HMWH-stimulated THP-1 and Jurkat or THP-1, Jurkat and HT cells, virtually all identified factors could be detected, additionally supplemented with IL-8 as a factor specifically occurring in cell line mixtures. This suggested that IL-16—as the only T cell-specific soluble mediator present under all MMP-9-inducing conditions—might be involved (either individually or in a combination with other mediators) in the induction of MMP-9 secretion from monocytes.

Interestingly, following stimulation with LMWH, no secreted cytokines/chemokines could be detected in co-cultures of THP-1 and Jurkat cells (data not shown) supporting the consideration that only HMWH (but not LMWH) is able to activate the production of MMP-9-inducing cytokine(s) from T cells.

### 2.7. Regulation of MMP-9 Expression in THP-1 by Jurkat-Derived IL-16 and sICAM-1

In the next step, THP-1 cells were stimulated with IL-16 to assess its influence on MMP-9 mRNA synthesis. Unexpectedly, this approach did not result in a significant upregulation of MMP-9, and equivalent negative results were obtained when THP-1 were stimulated with IL-13, MIF, or Serpin E1, i.e., the other factors found in the supernatant of HMWH-treated T cells. Only in the case of sICAM-1 a significant (but only approximately 3-fold) MMP-9 induction was observed (data not shown). This indicates that IL-16 alone is not the driver of MMP-9 production in monocytes and suggests that sICAM-1—potentially in combination with other secreted proteins—might contribute to the stimulatory effect on MMP-9 expression.

**Figure 6:**
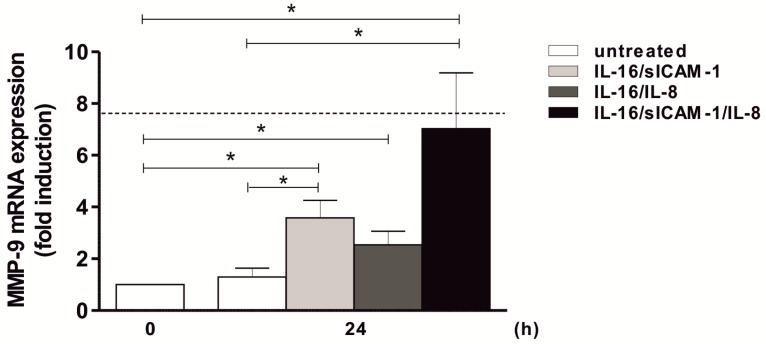
Induction of MMP-9 expression by IL-16, sICAM-1, and IL-8 in THP-1 cells. 2 × 10^6^ THP-1 cells/well were starved overnight. Afterwards, cells were stimulated with the mediators identified via Proteome Profiler Array: IL-16 and sICAM-1, IL-16 and IL-8, or IL-16, sICAM-1, and IL-8 for 24 h (5 ng/mL each). The dashed line indicates MMP-9 mRNA expression following TNF stimulation for 2 h as a positive control. MMP-9 mRNA expression was determined using qPCR (QuantiTect Custom Assay; housekeeping gene: GAPDH); mean ± SD, *n* = 3 (measured in duplicates). T test, * *p* ≤ 0.05.

To reveal whether an interaction between two or more factors is responsible for this effect, (T cell-specific) IL-16, sICAM-1 (due to its mild significant effect on MMP-9) and (cell mixture-specific) IL-8 were applied in different combinations. A slight but significant effect on MMP-9 was observed following stimulation of THP-1 cells with IL-16 and sICAM-1 or IL-16 and IL-8 with about 3– to 4-fold induction levels after 24 h (Figure 6). Our results suggest that the T cell-derived mediators IL-16 and sICAM-1 are acting in combination with the presumably monocyte-derived chemokine IL-8 thus mediating an induction of MMP-9. To further confirm these results, monocytes were incubated with the triple combination of these mediators. In fact, a synergistic influence of IL-16, sICAM-1, and IL-8 was confirmed (7-fold, i.e., as strong as the positive control TNF; Figure 6).

In contrast, the analysis of further cytokine combinations including MIF and IL-13 did not yield a significant increase in MMP-9 mRNA (data not shown) indicating that these proteins do not play an influential role in the regulation of MMP-9.

### 2.8. Regulation of IL-8 Expression in THP-1 by IL-16 and Autocrine Activation

**Figure 7:**
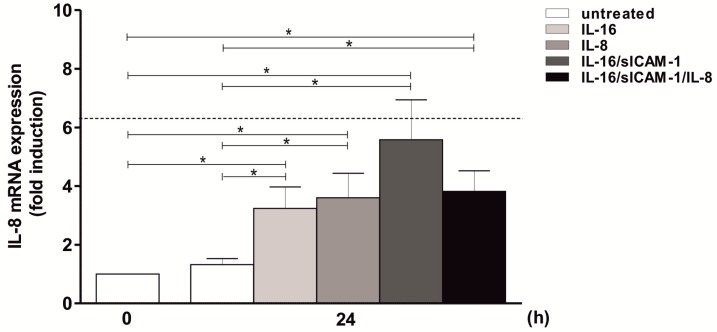
Induction of IL-8 expression by IL-16, sICAM-1, and IL-8 in THP-1 cells. 2 × 10^6^ THP-1 cells/well were starved overnight. Afterwards, cells were stimulated with IL-16, IL-8, IL-16 and sICAM-1, or IL-16, sICAM-1, and IL-8 for 24 h (5 ng/mL each). The dashed line indicates IL-8 mRNA expression following TNF stimulation for 2 h as a positive control. IL-8 mRNA expression was determined using qPCR (housekeeping gene: GAPDH); mean ± SD, *n* = 3 (measured in duplicates). T test, * *p* ≤ 0.05.

To further assess whether the supporting chemokine IL-8 is induced by the same mediator cocktail as MMP-9, or whether other factors are involved, monocytic cells were stimulated individually or with combinations of IL-16, sICAM-1, and IL-8 and the resulting IL-8 mRNA expression was measured. Initially, stimulation of THP-1 with individual factors as demonstrated for IL-16 and IL-8 itself resulted in a significant increase in the IL-8 measurement (3- to 4-fold induction; Figure 7). Moreover, further trials using IL-16 and sICAM-1 or IL-16, sICAM-1, and IL-8 also enhanced IL-8 expression with about 4- to 6-fold induction levels (thus roughly reaching a dimension also observed using the positive control TNF; Figure 7). With respect to the effect of IL-16, sICAM-1, and IL-8 on MMP-9 expression shown above, the results presented here substantiate the assumption that the T cell-derived factors IL-16 and sICAM-1 provoke the secretion of monocyte-derived IL-8. This combination, in turn, is able to induce both a continuing IL-8 production by monocytes and an increased MMP-9 expression.

## 3. Discussion

Like other MMPs, MMP-9 is a member of the broad ECM remodeling network [19,20] and plays a crucial role in physiological processes such as proliferation, differentiation, and wound healing, but also in tumor progression, metastasis, and angiogenesis [2,21] due to its ability to efficiently degrade (amongst others) gelatin, collagens (IV, V, VII, X, XIV), elastin, aggrecan, and fibronectin [22]. Its structure comprises an N-terminal pro-domain, the catalytically active metalloproteinase domain (including three fibronectin type II repeats and a zinc binding motif), and a hemopexin domain [20]. MMP-9 is initially synthesized as a latent pro-enzyme (the zymogen) which is activated via the so-called cysteine switch, i.e., the proteolytic cleavage of the pro-peptide [23]. To avoid deleterious effects in normal tissue, the enzymatic activity of MMP-9 is negatively regulated by tissue inhibitor of metalloproteinases (TIMP-) 3 and (to a lesser extent) 1, either by association with pro-MMP-9 or the inhibition of mature MMP-9 [24]. It has been reported that MMP-9 production is induced by TNF, interferon (IFN-) α, IL-1β, and several growth factors (e.g., epidermal growth factor (EGF), platelet-derived growth factor (PDGF)) [21]. Its mRNA expression is positively regulated via IκB kinase (IKK-), protein kinase C (PKC-), Ras-, or mitogen-activated protein kinases (MAPK; especially p38-, extracellular signal-regulated kinase (ERK-), and Jun N-terminal kinase (JNK-)) dependent signaling pathways, resulting in a nuclear factor κB (NF-κB-), specificity protein (Sp-) 1-, or activator protein (AP-) 1-driven gene expression. The repression of MMP-9 transcription is mainly Janus kinase (Jak)/signal transducer and activator of transcription (STAT-) dependent in response to IFN-β, IL-4, and IL-10 [21]. Consequently, MMP-9 expression is pharmacologically controlled using agents negatively affecting the MMP-9-regulating pathways or transcriptions factors, or both, thus especially reducing the activity of NF-κB or AP-1. For instance, MMP-9 expression can be significantly reduced via the suppression of NF-κB as demonstrated in breast cancer cells following the application of the isothiocyanate Sulforaphane [25] and in monocytic THP-1 cells treated with the labdane diterpenoid Andrographolide [26]. Consistently, MMP-9 can be induced in THP-1 cells using NF-κB-activating agents such as the synthetic triacylated lipopeptide Pam3CSK4 [27]. In a variety of other studies, the application of small molecule inhibitors for several kinases provoked a significant reduction of MMP-9 expression in different cell types. This has been demonstrated for inhibitors of PKC (e.g., RO318220, Calphostin C, Safingol), p38-MAPK (SB203580), MEK (PD098059), ERK (Rosiglitazone), and other signaling molecules (reviewed in [28]). Alternatively, signaling pathway activation can be prevented via neutralizing antibodies either against activating mediators (such as TNF), the respective receptors such as EGFR, or involved signaling molecules (e.g., MEK, Ras, PKC) [28].

In addition to the approaches to address MMP-9 as a target in neoplastic, vascular, and other diseases [5,29], it is also regarded as a promising diagnostic or prognostic factor, e.g., in inflammatory bowel disease [30] or stroke [7], rendering MMP-9 an interesting potential biomarker [30]. In the framework of a recently published study [8], we found a considerable relation between induction of MMP-9 expression and different anticoagulants used. Moreover, this relation affected both MMP-9 protein baseline values and kinetics, which was clearly shown in heparin samples with or without proteinase inhibitors. Consequently, the way of blood sampling influences the measurement of MMP-9. This is also reflected by further studies also showing that blood sampling influences the concentration and the diagnostic validity of MMP-9 [31] and other circulating MMPs (1, 2, 3, 7, and 8) [32]. Therefore, we aimed to characterize the effects of common anticoagulants on MMP-9 expression and to identify the cell types contributing to this effect to assess the impact of these anticoagulants in clinical applications and the suitability of MMP-9 as a biomarker. Our initial analyses showed that direct stimulation of THP-1, Jurkat, and HT cells (representing the major leukocyte cell types in the blood, i.e., monocytes, T cells, or B cells) with the common anticoagulants EDTA, citrate, or HMWH had no effect on the amounts of MMP-9 mRNA or protein. This indicates that its expression in blood cells is not induced directly by anticoagulants, but rather influenced via an indirect mechanism, i.e., an interaction of two or more cell types in the blood.

Based on this consideration, we performed an experiment with a mixture of the three cell lines and incubated them with EDTA, citrate, or HMWH. A strong (approximately 7-fold and 3.5-fold, respectively) and significant induction of MMP-9 mRNA and secreted protein in the mixture was only observed in response to HMWH, whereas EDTA and citrate had no effect on MMP-9 levels. These results are in good agreement to the literature since it has been reported that stimulation with heparin increases MMP-9 and TIMP levels in leukocytes and platelets [10], whereas citrate and EDTA appeared to be less effective [8]. We concluded that the MMP-9 expression in at least one of the included cell types depends on an activating interaction with another involved cell type in response to HMWH, either directly by cell-to-cell contacts or indirectly via the stimulation with a secreted mediator. To address this question, MMP-9 levels were analyzed in co-culture approaches including two different cell types each, i.e., THP-1/Jurkat, THP-1/HT, and Jurkat/HT. In contrast to all other combinations, stimulation of THP-1 and Jurkat cells with HMWH led to a significant increase in MMP-9 mRNA expression indicating that the interplay between monocytes and T cells is involved in the upregulation of MMP-9 in heparinized blood samples. The results may also reflect the tendency of leukocytes to generate the members of the MMP family not ubiquitously, but in distinct gene expression patterns [33] which has also been predicted bioinformatically for other cell types, e.g., fibroblasts [19].

In the next step, we analyzed whether the increased MMP-9 production depends on direct or indirect effects. With respect to the known ability of monocytes and macrophages to produce large amounts of MMP-9, e.g., in the course of tissue invasion [18], while T cells are important producers of cytokines [34], it was reasonable to speculate that during the interaction of monocytes and T cells, the latter are responsible for the secretion of one or more soluble mediator(s) to which monocytes react with increased MMP-9 production. Therefore, we measured the MMP-9 expression in THP-1 cells in response to stimulation with culture supernatant derived from HMWH-stimulated Jurkat cells. Indeed, a significant MMP-9 induction was observed under these conditions. In order to elucidate whether it is further possible to increase MMP-9 production under clinical-like circumstances, THP-1 were also incubated with the supernatant from Jurkat cells which have been activated by human plasma derived from heparin-containing monovettes. Again, even under these conditions, the MMP-9 mRNA amounts were enhanced in THP-1 cells stimulated with the transferred supernatant. Together, these results support the suggestion that in response to HMWH, T cells release monocyte-activating factor(s) while monocytes stimulated in this manner are the relevant producers of MMP-9. This assumption also fits to earlier reports demonstrating that monocytes are the main producers of MMP-9 among leukocytes [33].

**Figure 8:**
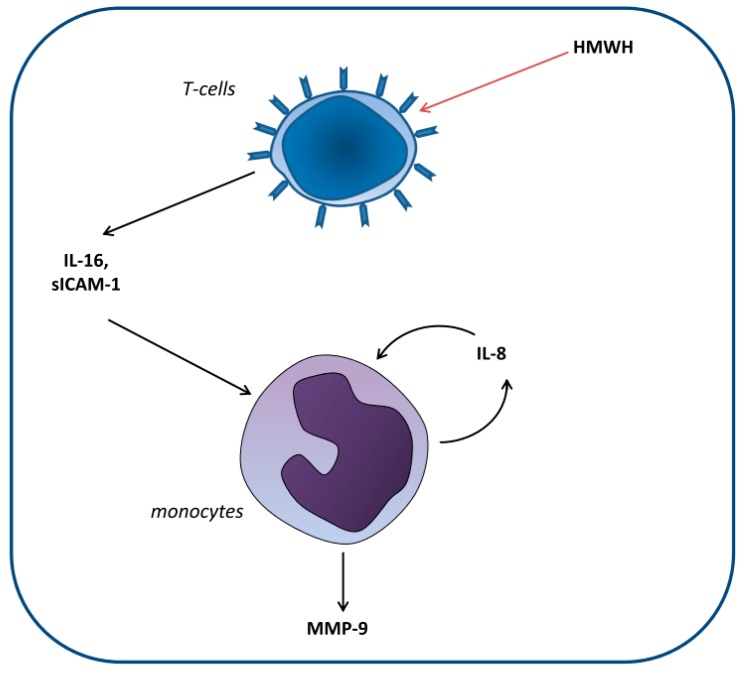
Proposed model of MMP-9 induction in monocytes by HMWH-treated T cells. Our data suggest that in response to HMWH, T cells secrete the mediators IL-16 and sICAM-1, which induce monocytic IL-8 production. Together, these factors yield a continuing IL-8 secretion as well as an enhanced MMP-9 production by monocytes.

To identify the T cell-derived mediator(s) involved in this process, cytokines and chemokines secreted by T cells in response to HMWH were monitored. These analyses revealed that HMWH-challenged Jurkat cells are characterized by the production of IL-13, IL-16, MIF, sICAM-1, and Serpin E1. Since most of these factors were also produced by THP-1 (MIF) or HT cells (IL-13, MIF, sICAM-1, and Serpin E1) in response to HMWH, IL-16 proved to be the only T cell-specific factor in this setting. Thus, IL-16, which is constitutively expressed but not secreted in T cells on the mRNA and pro-protein level [35], appeared to be a promising candidate cytokine, especially in consideration of other studies that have already shown that IL-16 is able to stimulate human monocytes, e.g., to increase the expression of pro-inflammatory cytokines [36]. A subsequent stimulation of THP-1 cells with IL-16, however, did not result in an enhanced MMP-9 expression, although its MMP-9 inducing capability has been demonstrated in vascular smooth muscle cells [37]. This suggested that further factors are required to achieve that effect. Among the mixtures of cytokines/chemokines then tested, only the combination consisting of IL-16, sICAM-1 (which showed a slight but significant effect on MMP-9 when individually applied), and IL-8 (which was the only mediator specifically expressed in co-culture experiments) exhibited a monocytic MMP-9 expression equivalent to the positive control (2 h TNF) and, more important, to that observed in the co-culture of THP-1 and Jurkat cells following HMWH stimulation. These circumstances indicated that T cell-derived IL-16 and sICAM-1, which had together a significant but relatively low effect on MMP-9, require the supporting presence of IL-8 to exploit their full activating potential on MMP-9. In this context, monocytes were the presumed source of IL-8, since they are known to produce large amounts of IL-8 in response to certain stimuli such as TNF [38]. Other factors, however, such as CCL5 or MIF appear not to be involved, presumably due to the absence of further stimulating agents, e.g., TNF or IL-1β, which have been reported to be necessary for their MMP-9-inducing activity [39,40]. To monitor whether IL-8 can be induced in monocytic cells by the same cocktail as MMP-9 (or at least one mediator included), THP-1 cells were stimulated individually or with combinations of IL-16, sICAM-1, and IL-8. Indeed, THP-1 cells expressed significantly increased amounts of IL-8 chemokine following a stimulation with IL-16 or IL-8 individually—thus confirming the previously reported autocrine regulation of IL-8 [41]—as well as combinations of IL-16 and sICAM-1 or IL-16, sICAM-1, and IL-8. In the latter cases, the IL-8 levels observed were roughly comparable to those found in the presence of the positive control TNF. Considered together with the result that IL-16, sICAM-1, and IL-8 are potent drivers of MMP-9 expression, our data suggest a model in which the presence of HMWH may lead to the secretion of T cell-derived IL-16 and sICAM-1 which induces monocytic IL-8 production. The combination of these factors appears to yield both a continuing IL-8 secretion as well as an enhanced MMP-9 production by monocytes (Figure 8). However, with respect to our experimental conditions, the participation of other cell types and further mediators could not be excluded and has to be addressed by future studies. It should also be mentioned that the mechanism presented here may also have an impact on the expression of further proteases thus potentially affecting their validity as biomarkers (such as MMP-2, -11, or -13 [42]).

In some of our experiments, TNF was used as a positive control in the cytokine stimulation experiments since it remarkably increases MMP-9 [43] and IL-8 expression [38] by monocytes. Thus, it appears surprising that the presence of HT cells had no influence on MMP-9 levels in THP-1- and HT-containing co-cultures although they secrete TNF in response to HMWH (Table 1). This might be ascribed to the absence of TNF in the case of the triple cell line culture. Whether this absence is due to an altered expression time course, ineffectively low TNF levels (i.e., under the detection limit of the proteome profiler membrane), or a suppression of HT-derived TNF expression in this cell mixture, remains to be established. In the pure THP-1/HT culture, in which TNF expression could be detected, the relatively low TNF amounts produced by HMWH-challenged HT cells (as represented by only slightly stained THF-representing dots on the detection membrane; data not shown) might be insufficient to evoke MMP-9 expression in THP-1 cells over time.

In contrast to HMWH, we did not detect a significant cytokine/chemokine secretion or MMP-9 expression in co-cultured THP-1 and Jurkat cells in response to LMWH. Although the effects of HMWH and LMWH on cytokine/chemokine or growth factor expression appear to differ among different conditions and cell lines [44], thus providing (at least in part) contradictory results, it has been observed that HMWH enhances the expression of CCL5 in endometrial carcinoma cells [44]. In heparinized plasma samples, elevated levels of methyl-accepting chemotaxis protein (MCP), stem cell factor (SCF), PDGFβ, vascular endothelial growth factor (VEGF), CXC chemokine ligand (CXCL) 11, and Serpin E1 [45] or IL-6, -8, -17, granulocyte/macrophage-colony stimulating factor (GM-CSF), and macrophage inflammatory protein (MIP-) 1 in comparison to citrate or EDTA plasma, or both, were detected [46]. In contrast, LMWH has been reported to significantly reduce the expression of pro-inflammatory cytokines and chemokines in several cell types. For instance, LMWH application inhibited the secretion of IL-4 and TNF by mast cells [47] and the expression of IL-6 and -8 in epithelial cells [48] as well as IL-4, -5, -13, and TNF in T cells, effects that fit well to the anti-inflammatory features of LMWH [49]. The remarkable difference in cytokine induction between HMWH and LMWH described in the literature and observed in our study may be attributed to the molecular structure of the respective heparins. Natural heparin is exclusively produced by mast cells and predominantly isolated on an industrial scale from porcine intestinal mucosa [50]. Both the unfractionated heparin (i.e., HMWH) and its fractionated derivative LMWH (produced by chemical or enzymatic processing of HMWH [50]) are glycosaminoglycans exhibiting a common molecular structure, i.e., repeating disaccharide units consisting of variably sulfated glucosamine, glucuronic acid, or iduronic acid [51]. Due to the general structural similarity, the anticoagulant mechanism is comparable: both heparins interact with antithrombin and suppress the activity of factor Xa and—to a lesser extent—thrombin [50]. However, in contrast to the long-chained HMWH with an average molecular weight of 16 kDa, LMWH is characterized by considerably shorter disaccharide chains resulting in an average molecular weight of 3.5–6 kDa [51], a reduced charge per chain, and less unspecific interactions with proteins [52]. In consequence, HMWH’s length, charge, and tendency towards unspecific interactions may play a crucial role in the activation of T cells. For instance, an interaction with specific surface receptors is to be assumed, since it has been shown that unfractionated heparin is able to interact with the high mobility group box 1 protein receptor on macrophages [53] as well as G-protein-coupled receptor GPR56 on a variety of human cell lines [54]. Moreover, in the case of EDTA and citrate, the—in comparison to HMWH—negligible length and charge may be crucial for the absence of an MMP-9-inducing effect of these anticoagulants under the conditions tested in our study. Taking these points into account, one can speculate that receptor crosslinking may play a role during HMWH-induced activation of T cells, an effect which could not be provided by LMWH, EDTA, or citrate.

Our results also possess some clinical implications. For instance, HMWH-containing monovettes should be avoided when MMP-9 levels have to be assessed as a biomarker to circumvent its overvaluation. More important, heparin is also administered systemically during therapeutic approaches, e.g., for the treatment of venous thromboembolism (VTE) [13] or the prevention of myocardial infraction during the acute phase of unstable angina [55]. Known tissue-damage-associated adverse effects of heparin such as increased tendency towards bleeding [56] may be influenced or intensified by its MMP-9 promoting capacity. LMWH which according to our data does not appear to possess an influence on the expression of MMP-9 nor any cytokine/chemokine monitored may represent a preferable “gentle” alternative to HMWH. In another study, even a reduction of MMP-9 concentration and activity has been reported in the plasma of LMWH-treated patients with abdominal aortic aneurysm [57], an effect which might be connected to the LMWH-dependent downregulation of IL-8 as shown in enoxaparin-treated epithelial cells [48]. Accordingly, LMWH is increasingly used as an anticoagulant, e.g., for VTE prevention [58,59].

In summary, our study suggests that the increased MMP-9 expression previously found in HMWH blood samples may be ascribed to an HMWH-induced secretion of IL-16 and sICAM-1 by T cells resulting in an increased production of MMP-9 by monocytes, a process potentially supported by IL-8 in an autocrine feedback loop (Figure 8). This implies that HMWH shall be used cautiously in clinical applications.

## 4. Materials and Methods

### 4.1. Collection and Storage of Human Heparin Plasma

A total of three venous blood samples from healthy individuals were collected at the Department of Clinical Chemistry at Hannover Medical School. Informed consent was obtained from all donors before blood sampling. The experiments were carried out in accordance with the relevant guidelines and regulations and approved by the Hannover Medical School ethics committee in accordance with the Declaration of Helsinki (No. 388-2008, 02.12.2008). Blood sampling was performed using commercially available heparin- (i.e., HMWH) containing monovette tubes (Sarstedt, Nümbrecht, Germany). Blood samples were immediately centrifuged at 1900× *g* for 10 min and after removal of the supernatant, plasma samples were aliquoted and stored at −80 °C until assay.

### 4.2. Cell Lines and Cell Culture Conditions

For the analyses, THP-1 (human acute monocytic leukemia cell line; ACC-16), Jurkat (human T cell leukemia cell line, ACC-282), and HT cells (human B cell lymphoma cell line, ACC-567) were used (DSMZ, Braunschweig, Germany), representing monocytes, T cells, and B cells, respectively. All cell lines were cultured in RPMI 1640 medium containing 300 mg/L l-glutamine (PAA, Linz, Austria), supplemented with 10% fetal calf serum (FCS), 100 U/mL penicillin, and 100 mg/mL streptomycin (Biochrom, Berlin, Germany) at 37 °C in a humidified atmosphere containing 5% CO_2_ [60].

Cell culture experiments were performed in 6-well plates (Sarstedt) with 2 mL medium/well. For stimulation experiments, cells were incubated without antibiotics. For starvation, the FCS concentration in the culture medium was reduced to 1% and cells were incubated overnight. Starved cells without further treatment/stimulation served as negative controls to avoid FCS-induced disturbances of the respective measurements.

### 4.3. Cell Culture Experiments

#### 4.3.1. Individual and Co-Culture Experiments

In the initial experiments, 2 × 10^6^ THP-1, Jurkat, or HT cells/well were starved in individual cultures overnight. Afterwards, cells were stimulated with 3.2 mg EDTA (Sigma Aldrich, Darmstadt, Germany), 10 µL HMWH (=50 IU; Ratiopharm, Ulm, Germany) or LMWH (Clexane; Sanofi-Aventis, Frankfurt, Germany or Fragmin; Pfizer, Berlin, Germany), or 220 µL citrate (Sarstedt) per well and harvested after 0, 4, 6, and 24 h for the analysis of MMP-9 mRNA expression.

In double co-culture experiments, THP-1 and Jurkat, THP-1 and HT, or Jurkat and HT cells were cultivated together (in total 2 × 10^6^ cells/well, i.e., 1 × 10^6^ cells per cell line). Following starvation overnight, cells were stimulated with the respective anticoagulants and harvested at the indicated time points. In addition, supernatants of the respective double co-cultures were collected for the analysis of secreted proteins.

Furthermore, in a triple co-culture experiment a mixture of the three cell lines used was seeded with 2.1 × 10^6^ cells/well (i.e., 0.7 × 10^6^ cells per cell line) and starved overnight. Then, the respective anticoagulant was added and the cells were harvested and the supernatant collected at the indicated time points.

#### 4.3.2. Cell Supernatant Transfer Experiments

In a further series of experiments, starved monocytic THP-1 cells were stimulated with the supernatant of anticoagulant-treated Jurkat or HT cells and the MMP-9 expression was analysed as indicated. Alternatively, starved THP-1 cells were incubated with supernatant from starved Jurkat or HT cells which have been stimulated for 24 h with human plasma derived from blood samples collected in HMWH-containing monovettes.

#### 4.3.3. Cytokine/Chemokine Stimulation Experiments

Following a starvation phase overnight, 2 × 10^6^ THP-1 cells/well were stimulated with 5 ng/mL MIF, 5 ng/mL IL-13, 5 ng/mL IL-16, 5 ng/mL sICAM-1, 25 ng/mL Serpin E1, or 5 ng/mL IL-8 (Peprotech, Hamburg, Germany), either individually or in combination, for 24 h. The respective concentrations were derived from the respective product sheet by selecting a low effective concentration and then validated by dose response experiments. As a positive control, starved THP-1 cells were stimulated for 2 h with 25 ng/mL human TNF (Sigma Aldrich). Following stimulation, cells were harvested and the MMP-9 or IL-8 mRNA expression was measured.

### 4.4. RNA Extraction, cDNA Synthesis, and qPCR

Cells were lysed and RNA was isolated using the RNeasy Mini Kit (Qiagen, Hilden, Germany) according to the manufacturer’s instructions [61]. RNA was eluted in 50 μL RNase-free water and concentrations were determined using the NanoDrop ND-1000 photometer at 260/280 nm (Peqlab, Erlangen, Germany). For first-strand complementary DNA (cDNA) synthesis, 1 μg total RNA per sample were reverse transcribed using the SuperScript™ II Reverse Transcriptase kit (Invitrogen, Carlsbad, CA, USA) and 250 ng random primers in a total volume of 100 μL as previously described [62]. Following reverse transcription, cDNA samples were immediately frozen and stored at −80 °C until further use. Quantitative polymerase chain reaction (qPCR) was performed using the LightCycler 480 II (Roche Diagnostics, Mannheim, Germany) as described in [8]. In short, MMP-9 analyses were based on the QuantiTect^®^ Custom Assay (Qiagen; forward primer: 5′-TCCAGTACCGAGAGAAAG-3′, reverse primer: 5′-CAGGATGTCATAGGTCACGTAG-3′, hybridization probe: 5′-non-fluorescent quencher-GGAGTGAGTTGAACCAG-6-carboxyfluoroscein-3′). Suitability of glyceraldehyde-3-phosphate dehydrogenase (GAPDH), actin, and β2-microglobulin as housekeeping genes was assessed in 5 randomly selected cDNA samples. In all cases, expression levels were roughly comparable among tested samples. GAPDH was finally selected as housekeeping gene since it exhibited the highest expression levels. GAPDH mRNA determination was performed using sequence-specific fluorescent resonance energy transfer probes (forward primer: 5′-TGCTGAGTATGTCGTGGAGTC-3′, reverse primer: 5′-GGATGCAGGGATGATGTTCT-3′; donor hybridization probe: 5′-GACAACTTTGGTATCGTGGAAGGACTCATGACCACA-fluorescein thiocyanate-3′, acceptor hybridization probe: 5′-Cy5.5-CTGAGCGTGGCTATTCCTTCGTGACTACTG-phosphate-3′). 5 μL cDNA were mixed with 4 pmol hybridization probes, 10 pmol forward and reverse primers, and 10 μL LightCycler 480 Probes Master (Roche). H_2_O was added to a final volume of 20 μL. PCR conditions were selected as follows: initial hot start incubation (95 °C, 5 min), denaturation (95 °C, 10 s), annealing (56 °C, 30 s for MMP-9; 59 °C, 30 s for GAPDH), and extension (72 °C, 30 s).

IL-8 mRNA expression was determined using the SYBR Select Master Mix (Invitrogen) and the LightCycler 480 (forward primer: 5′ -TCCTGTTTCTGCAGCTCTGG-3′, reverse primer: 5′ -GGCCACTCTCAATCACTCTC-3′). An amount of 5 μL cDNA was mixed with 10 pmol forward and reverse primers, and 10 μL SYBR Select Master Mix. H_2_O was added to a final volume of 20 μL. PCR conditions were selected as follows: enzymatic degradation of contaminating uracil-containing DNA (50 °C, 2 min), initial hot start incubation (95 °C, 2 min), denaturation (95 °C, 10 s), annealing (59 °C, 15 s) extension (72 °C, 20 s) [38].

For the calculation of cDNA concentration, plasmid standards were generated and used in serial dilutions ranging from 1 mg/μL to 100 fg/μL as previously described [63].

### 4.5. Enzyme-Linked Immunosorbent Assay

Measurement of total secreted MMP-9 protein in the cell culture supernatant was performed using the Quantikine Kit (R&D Systems, Wiesbaden, Germany). Amounts of secreted total MMP-9 protein were assessed in thawed cell culture supernatant (stored at −150 °C until use) according to the manufacturer’s instructions as described before [8]. The mean intra- and inter-assay coefficients of variation were given as 2.3% and 7.5%, respectively. The minimum detectable concentration was given as 0.156 μg/L.

### 4.6. Proteome Profiler Array

To identify secreted mediators, the Proteome Profiler Human XL Cytokine Array (based on the sandwich ELISA principle; R&D Systems) was carried out according to the manufacturer’s instructions [64]. In short, an array membrane (consisting of capture antibodies spotted in duplicate on nitrocellulose) was incubated with 1.5 mL diluted supernatant (derived from HMWH-stimulated individual cell lines or co-cultures) and a cocktail of biotin-coupled capture antibodies (15 µL) overnight at 4 °C. Afterwards, the membrane was washed and incubated with 1.5 mL Streptavidin-HRP dilution for 30 min. Subsequently, membrane-bound cytokines/chemokines were detected using the chemiluminescent detection reagent and Amersham Hyperfilm ECL films (GE Healthcare, Freiburg, Germany).

### 4.7. Statistical Analysis

Data were described as means ± standard deviation (SD). The Kruskal–Wallis test was applied to analyze differences in mRNA or protein levels among several time points. The unpaired T test was used to analyze differences among mRNA levels following LMWH or cytokine stimulation. Analyses were performed using IBM SPSS Statistics, version 24 (IBM Deutschland GmbH, Ehningen, Germany).

## Figures and Tables

**Table 1:** Cytokines/chemokines expressed by HMWH-treated THP-1, Jurkat, and HT cells as determined by Proteome Profiler Human XL Cytokine Array. T cell-specific IL-16 and co-culture-specific IL-8 are highlighted in bold.

Cell Line(s)	Identified Cytokines/Chemokines
THP-1	IL-1RA, CCL5, MIF
Jurkat	MIF, IL-13, **IL-16**, sICAM-1, Serpin E1
HT	MIF, IL-13, sICAM-1, Serpin E1, TNF
THP-1, Jurkat, and HT	IL-1RA, CCL5, MIF, IL-13, **IL-16**, sICAM-1, Serpin E1, **IL-8**
THP-1 and Jurkat	IL-1RA, CCL5, MIF, IL-13, **IL-16**, sICAM-1, Serpin E1, **IL-8**

## Abbreviations

AP	Activator protein
cDNA	Complementary DNA
CCL	CC-chemokine ligand
CXCL	CxC-chemokine ligand
DSMZ	Deutsche Sammlung von Mikroorganismen und Zellkulturen
ECL	Enhanced chemiluminescence
ECM	Extracellular matrix
EDTA	Ethylenediaminetetraacetic acid
EGF	Epidermal growth factor
ELISA	Enzyme-linked immunosorbent assay
ERK	Extracellular signal-regulated kinase
FCS	Fetal calf serum
GAPDH	Glyceraldehyde-3-phosphate dehydrogenase
GM-CSF	granulocyte/macrophage-colony stimulating factor
GPR	G-protein-coupled receptor
HMGB	High mobility group box
HMWH	High-molecular-weight heparin
HRP	Horseradish peroxidase
IFN	Interferon
IKK	IκB kinase
IL	Interleukin
IL-1RA	IL-1 receptor antagonist
IU	International unit
Jak	Janus kinase
JNK	Jun N-terminal kinase
LMWH	Low-molecular-weight heparin
MAPK	Mitogen-activated protein kinasesmethyl-accepting chemotaxis protein
MCP	Methyl-accepting chemotaxis protein
MIF	Macrophage migration inhibitory factor
MIP	Macrophage inflammatory protein
MMP	Matrix metalloproteinase
NF-κB	Nuclear factor κB
PDGF	Platelet-derived growth factor
PKC	Protein kinase C
qPCR	Quantitative polymerase chain reaction
SCF	Stem cell factor
SD	Standard deviation
sICAM	Soluble intercellular adhesion molecule
Sp	Specificity protein
STAT	Signal transducer and activator of transcription
TIMP	Tissue inhibitor of metalloproteinases
TNF	Tumor necrosis factor
VEGF	Vascular endothelial growth factor
VTE	Venous thromboembolism
